# The “State of Implementation” Progress Report (SIPREP): a pilot demonstration of a navigation system for implementation

**DOI:** 10.1186/s43058-020-00085-7

**Published:** 2020-11-05

**Authors:** Edward J. Miech, Angela Larkin, Julie C. Lowery, Andrew J. Butler, Kristin M. Pettey, Nicholas A. Rattray, Lauren S. Penney, Jennifer Myers, Teresa M. Damush

**Affiliations:** 1 VA Precision Monitoring to Transform Care (PRIS-M), Quality Enhancement Research Initiative, HSR&D, Richard L. Roudebush VA Medical Center, Mail Code 11H, 1481 West 10th Street, Indianapolis, IN 46202 USA; 2 VA Center for Clinical Management Research, VA Ann Arbor Healthcare System, 2215 Fuller Road, Ann Arbor, MI 48105 USA; 3 School of Health Professions, University of Alabama at Birmingham, Dean’s Office, SHPB 630, 1716 9th Avenue South, Birmingham, AL 35294 USA; 4 VA Southeast Network Office (VISN 7), 3700 Crestwood Parkway, NW, Suite 500, Duluth, GA 30096-5585 USA; 5 The Elizabeth Dole Center of Excellence for Veteran and Caregiver Research, South Texas Veterans Health Care System, 7400 Merton Minter, San Antonio, TX 78229 USA

**Keywords:** Implementation process, Implementation stage, Rapid-cycle evaluation, Demonstration project, Program evaluation, Implementation strategy, Data visualization

## Abstract

**Background:**

Implementation of new clinical programs across diverse facilities in national healthcare systems like the Veterans Health Administration (VHA) can be extraordinarily complex. Implementation is a dynamic process, influenced heavily by local organizational context and the individual staff at each medical center. It is not always clear in the midst of implementation what issues are most important to whom or how to address them. In recognition of these challenges, implementation researchers within VHA developed a new systemic approach to map the implementation work required at different stages and provide ongoing, detailed, and nuanced feedback about implementation progress.

**Methods:**

This observational pilot demonstration project details how a novel approach to monitoring implementation progress was applied across two different national VHA initiatives. Stage-specific grids organized the implementation work into columns, rows, and cells, identifying specific implementation activities at the site level to be completed along with who was responsible for completing each implementation activity. As implementation advanced, item-level checkboxes were crossed off and cells changed colors, offering a visual representation of implementation progress within and across sites across the various stages of implementation.

**Results:**

Applied across two different national initiatives, the SIPREP provided a novel navigation system to guide and inform ongoing implementation within and across facilities. The SIPREP addressed different needs of different audiences, both described and explained how to implement the program, made ample use of visualizations, and revealed both what was happening and not happening within and across sites. The final SIPREP product spanned distinct stages of implementation.

**Conclusions:**

The SIPREP made the work of implementation explicit at the facility level (i.e., who does what, and when) and provided a new common way for all stakeholders to monitor implementation progress and to help keep implementation moving forward. This approach could be adapted to a wide range of settings and interventions and is planned to be integrated into the national deployment of two additional VHA initiatives within the next 12 months.

Contributions to the literature

- Implementation of new programs within healthcare systems can be extraordinarily complex, occurring under conditions that can change rapidly and unexpectedly, where individuals within the same healthcare organization can have different perspectives on how implementation unfolds, and where local context can vary widely.
- In a pilot demonstration spanning two national VA initiatives, the SIPREP offered a systematic and comprehensive way to map the implementation work required at different stages of the implementation process and provided ongoing, detailed, and nuanced feedback about implementation progress over time across multiple facilities.
- As a novel approach to navigating the process of implementing new programs across multiple sites, the SIPREP makes the work of implementation explicit at the facility level (i.e., who does what, and when) and provides a new, dynamic approach for local staff, national program team members, and operational partners to pinpoint areas where extra support and intervention might be focused to help keep implementation moving forward.

## Background

Implementation of new programs within healthcare systems can be extraordinarily complex, unfolding differently across sites due to variation in local context and conditions [1–3]. A structured approach to capturing implementation progress that helped organize and manage this complexity could play an important role in supporting and improving active implementation. A prospective and longitudinal approach could also help maintain accuracy in the reporting of implementation-related information while minimizing retrospective recall, which can introduce memory and hindsight bias [4].

Several approaches already in use in health services relate to the systematic capture and reporting of implementation-related work. For example, rapid evaluation and assessment methods (REAM) have long offered a way for researchers in diverse settings to collect and analyze data on an accelerated timetable while retaining rigor [5–7]. Matrix displays integrate and organize large amounts of data in rows and columns that can be easily sorted and sifted and offer a visual method to support the identification of emergent themes and findings [8, 9]. The Stages of Implementation Completion (SIC) is a validated instrument that maps key implementation tasks across eight stages and tracks progress through completion dates [10]. “Implementation playbooks” provide users with detailed guides or blueprints about how to implement programs, along with practical tips and resources [11–13].

Building on these prior approaches, a team of implementation scientists sought to develop a new system to capture and report implementation progress across time and space in ways that supported active implementation of new programs at multiple sites. We conducted a demonstration evaluation of two different applications to determine the feasibility of this approach.

## Methods

In 2016, the Precision Monitoring (PRIS-M) QUERI (Quality Enhancement Research Initiative) based at the Roudebush VA Medical Center in Indianapolis, Indiana, was charged with supporting and studying the implementation of the VHA Tele-Stroke Robotic Rehabilitation program at four pilot sites around the USA. The 7-person implementation team based in Indianapolis included three doctoral-level implementation scientists who collectively had been working in implementation science for over 30 years in VHA, a senior physician-researcher, a masters-level program manager, and a research assistant.

As part of this work, the implementation support team developed general specifications of a new “State of Implementation” Progress Report (SIPREP) over a 6-month period in 2018. They drew upon multiple sources of information to map the implementation work: weekly national program phone calls, discussions with individual participants, site visits, notes from implementation team meetings, and online resources. Stage-specific grids organized the implementation work into columns, rows, and cells, identifying specific implementation activities to be completed; who was responsible for completing each implementation activity; and the timing/level of each activity (early/basic, intermediate, or late/advanced). Additional links provided access to specific tips and resources that could assist local staff in completing particular implementation activities.

The SIPREP was subsequently applied independently by two different teams working on two different and unrelated national VHA QI initiatives. The first initiative was the Tele-Stroke Robotic Rehabilitation program based at the Atlanta VA Medical Center. The Tele-Stroke Robotic Rehabilitation program provided rural veterans recovering from a stroke with an innovative, in-home solution for physical rehabilitation that especially benefited veterans living in rural areas distant from Veterans Health Administration medical centers. The program was a quality improvement (QI) project funded by the VA Office of Rural Health as an Innovations Project to be implemented at four pilot sites.

In FY17, the PRIS-M implementation support team assisted the Atlanta-based clinical team with implementation of the program and was specifically charged with providing ongoing feedback to the Tele-Stroke Robotic Rehabilitation program about implementation progress at the participating sites as well as providing guidance for any future scaling up of the program if it received approval for a larger rollout. With these aims in mind, the team developed the new “State of Implementation” Progress Report (SIPREP) approach.

Each of the four participating VA medical centers was given its own designated grid for each stage of implementation. Within each grid, item-level checkboxes were checked off and cells changed colors as particular activities were completed, offering a visual representation of implementation progress within and across sites across the various stages of implementation. The SIPREP was hosted on a VA SharePoint platform, and the implementation support team created, maintained, and updated the SIPREP for all four VA medical centers.

Two key concepts used to organize the SIPREP were “milestones” and “stages.” Milestones were significant implementation achievements that occurred in a chronological order. In the implementation of the tele-robotics program, there were five milestones: initial agreement to participate; kickoff; enrolling 1st patient; enrolling 10th patient; and adoption/sustaining. Stages involved from getting from one milestone to the next.

Stage-specific “grids” organized the implementation work into columns, rows, and cells, identifying specific implementation activities to be completed; who was responsible for completing each implementation activity; and the timing and level of each activity. Grid columns specifying who completed particular implementation activities, and grid rows indicated the timing/level of each activity.

Additional links provided access to specific tips and resources that could assist local staff in completing particular implementation activities.

Each of the four participating VA medical centers was given its own designated grid for each stage of implementation. Within each grid, item-level checkboxes were checked off and cells changed colors as particular activities were completed, offering a visual representation of implementation progress within and across sites across the various stages of implementation.

A different implementation support team located in a different part of the USA independently applied the SIPREP to evaluate implementation progress on another national VHA program. The PeRsonalizing Options for Veteran Engagement (PROVE) QUERI program based at the VA Ann Arbor Healthcare System began using the SIPREP as part of implementing and evaluating a web-based, provider-facing tool for enhancing shared decision-making with patients eligible for lung cancer screening.

The Lung Decision Precision (LDP) tool was initially implemented using a six-month, virtual quality improvement training approach in four VA medical centers beginning in 2017. Four other VA medical centers served as a control group, in which the tool was implemented using a one-time provider education approach.

In 2018, midway through implementation of LDP across the eight participating sites, the PROVE QUERI lead investigator learned about the SIPREP from a VHA webinar series and discussed potential use of the tool with the project manager in charge of the LDP implementation. The eight participating sites were all progressing with implementation at different rates and had implemented lung cancer screening in different ways, which affected their interest in and ability to use LDP. As a result, it was challenging for the national team in Ann Arbor to keep track of implementation progress at individual sites. The lead investigator thought the SIPREP would be an apt mechanism for capturing the status of implementation at each site and clearly delineating next steps.

## Results

Similar to how a navigation system works in a moving vehicle, the SIPREP as a general approach offered a dynamic, telescoping view of implementation progress that was capable of being big-picture or ultra-granular; oriented users as to current position; showed what loomed ahead; and provided detailed options for how to get to the next destination. The SIPREP offered multiple color-coded, single-page visualizations of implementation progress both within and across sites as well as within and across stages.

In the VHA Tele-Stroke Robotic Rehabilitation project, the SIPREP allowed for “assessment at a glance” of the progress of an individual site across all four implementation stages.

**Fig. 1:**
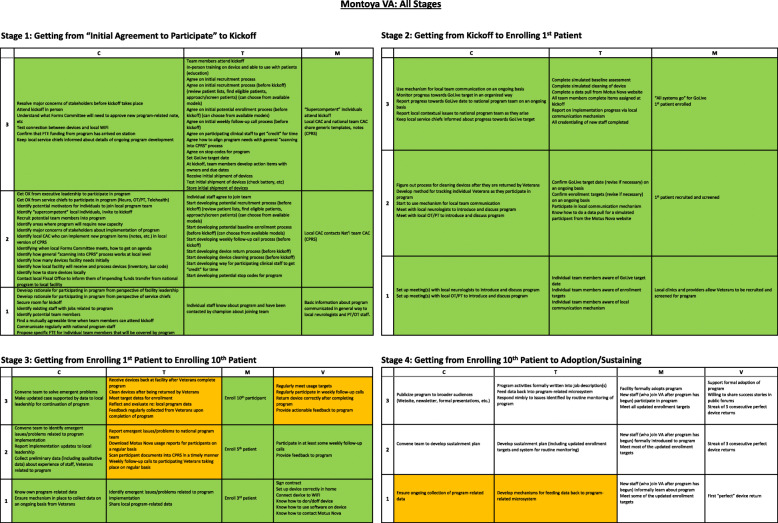
Implementation progress across all four stages of implementation at a single medical center for the Tele-Stroke Robotic Rehabilitation project

Figure 1 displays the implementation progress of one medical center—the “Montoya VA”—across all four stages. Within each grid, implementation support team changed cell colors as particular activities were completed, offering a visual representation of implementation progress. Green indicates “completion”; orange indicates “in progress”; white/blank indicates “not yet started.” This visualization also shows flexibility in the SIPREP in that a facility could work on activities in a more advanced stage even if all activities in an earlier stage have not yet been completed; unfinished activities from an earlier stage can be completed at a later point in time. Each “link” is a hyperlink providing immediate connections to specific grids or cells.

**Fig. 2:**
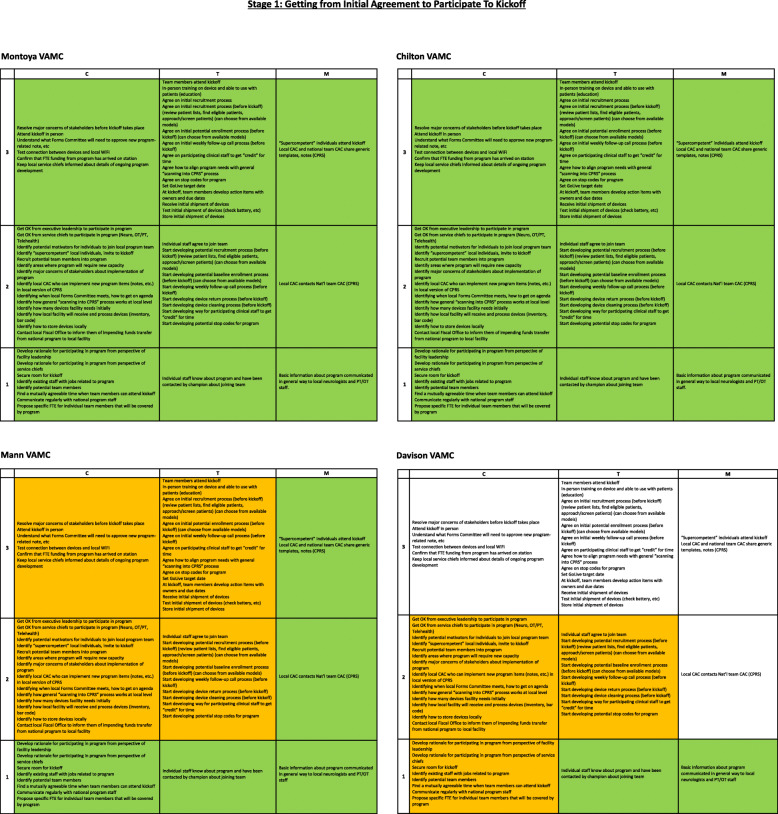
Implementation progress across four sites for stage 1 of implementation for the Tele-Stroke Robotic Rehabilitation project

Figure 2 displays the implementation progress of all four medical centers for stage 1 at one point in time. This view shows substantial variation in implementation progress across sites. The grids for the Montoya and Chilton VA medical centers are solid green, as they both have completed all implementation activities. The grid for the Mann VA medical center is about half green and half orange, indicating it has completed about half of the implementation work in the stage. The grid for the Davison VA medical center is largely white/blank, indicating that little implementation work has been undertaken so far. This visualization shows how the SIPREP made the work of implementation explicit at the facility level (i.e., who does what, and when) and provided a new way for VHA participants, national program team, and operational partners to understand what was happening—and not happening—at the facility level, pinpointing areas where extra support and intervention might be focused to help keep implementation moving forward.

These visualizations only represent a small subset of possible views within the SIPREP Tele-Stroke Robotic Rehabilitation project. In the actual SIPREP, a nested relationship dynamically connected grids, cells, and items via hyperlinks. Users could zoom in or out at will with a progression from a birds-eye view of all sites and all stages of implementation all the way to the level of a single item, in effect unifying five different implementation resources in one place: a how-to manual, a knowledge base, an implementation progress report, a diagnostic tool, and a focused checklist [14].

**Fig. 3:**
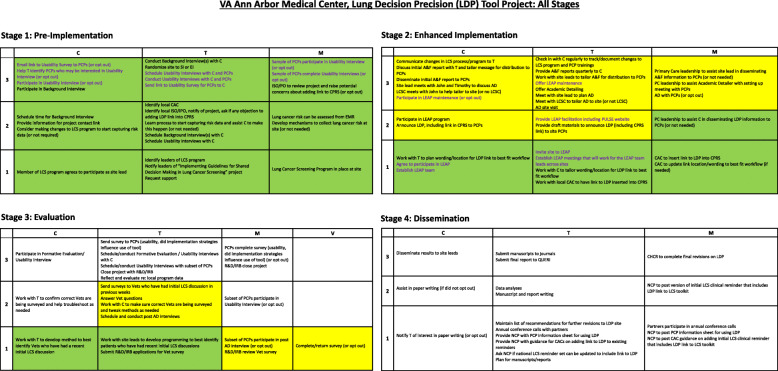
Four stages of implementation work for the Lung Decision Precision (LDP) tool project

In the second project, the LDP implementation support team used four milestones in their application of the SIPREP: pre-implementation, enhanced implementation, evaluation, and dissemination. The SIPREP allowed for updated visualizations of differences in implementation status across the different project arms, saving time on email updates and meeting agendas. Figure 3 shows how the LDP implementation support team used the SIPREP system to map out across four stages the facility-level implementation work needed to put the Lung Decision Precision (LDP) tool project into practice.

To assist in documentation, the LDP team also added a “Project History” feature to their version of the SIPREP which allowed the implementation support team to document information including individuals involved and completion dates related to the local accomplishment of specific implementation activities.

## Discussion

In this cross-initiative pilot demonstration project, the SIPREP navigational system was successfully applied across two different VHA initiatives by independent VHA implementation support teams. Like a map application on a phone or a navigation system in a moving vehicle, the SIPREP offered telescoping perspectives on implementation progress, ranging from the macro-perspective of a “birds-eye view” to the micro-perspective of a “street view.”

The format of the SIPREP was influenced by the prior experience of the implementation team members in using matrix displays and applying rapid evaluation and assessment methods on earlier QUERI projects, including the Prospectively-Reported Implementation Update and Score (PRIUS) [15]. The “SIPREP” name itself is a nod to the term “sitrep,” shorthand in intelligence, planning, military, and emergency response contexts for a “situation report” that captures salient “on the ground” information about the current state of affairs in a particular setting [16].

The SIPREP approach seems to be highly adaptable to a wide range of settings and interventions and is already planned to be integrated into the national deployment of additional national VHA initiatives within the next 12 months. As part of this initiative, work is now underway to develop a web-based SIPREP application that allows different users to customize the SIPREP for individual projects. Once it is field-tested, the SIPREP application will be made broadly and freely available both within and outside the VHA healthcare system; more information on accessing the SIPREP application can be found at https://www.siprep.net.

### Limitations

The SIPREP system has been used so far to support active implementation in two national VHA initiatives. It may not generalize to other projects or other healthcare contexts substantially different from these two examples. Using the SIPREP system during project implementation requires additional time and resources on the part of national implementation support teams as well as participating sites, and this information was not collected as part of this pilot demonstration project; future studies of the SIPREP should consider capturing cost data. This demonstration project employed an observational design, whereas a trial would be necessary to ascertain the relative effectiveness of the SIPREP.

## Conclusions

As a novel approach to mapping implementation progress, the SIPREP made the work of implementation explicit at the facility level (i.e., who does what, and when) and provided a new way for facility-level staff, national program team members, and operational partners to understand what was happening—and not happening—at the facility level, pinpointing areas where extra support and intervention might be focused to help keep implementation moving forward. The SIPREP approach was dynamic and prospective and addressed the different needs of different audiences. As a navigation system for implementation, the SIPREP appears to offer original insights and actionable recommendations to users that support the dynamic and complex process of implementation within healthcare settings.

## Data Availability

Data generated during this pilot demonstration project are included in this published article. An earlier and longer version of this article that features additional visualizations is available in a preprint format [14].
