# Correction to: The “State of Implementation” Progress Report (SIPREP): a pilot demonstration of a navigation system for implementation

**DOI:** 10.1186/s43058-020-00095-5

**Published:** 2020-12-03

**Authors:** Edward J. Miech, Angela Larkin, Julie C. Lowery, Andrew J. Butler, Kristin M. Pettey, Nicholas A. Rattray, Lauren S. Penney, Jennifer Myers, Teresa M. Damush

**Affiliations:** 1 VA Precision Monitoring to Transform Care (PRIS-M), Quality Enhancement Research Initiative, HSR&D, Richard L. Roudebush VA Medical Center, Mail Code 11H, 1481 West 10th Street, Indianapolis, IN 46202 USA; 2 VA Center for Clinical Management Research, VA Ann Arbor Healthcare System, 2215 Fuller Road, Ann Arbor, MI 48105 USA; 3 School of Health Professions, University of Alabama at Birmingham, Dean’s Office, SHPB 630, 1716 9th Avenue South, Birmingham, AL 35294 USA; 4 VA Southeast Network Office (VISN 7), 3700 Crestwood Parkway, NW, Suite 500, Duluth, GA 30096-5585 USA; 5 The Elizabeth Dole Center of Excellence for Veteran and Caregiver Research, South Texas Veterans Health Care System, 7400 Merton Minter, San Antonio, TX 78229 USA

**Correction to: Implement Sci Commun 1, 102 (2020)**

**https://doi.org/10.1186/s43058-020-00085-7**

Following publication of the original article [1], it was reported that the incorrect version of a reviewer’s comments were published. The correct version has now been uploaded and the original article has been corrected.
